# The R.M.O. and outside Emergency Calls

**Published:** 1909-01-02

**Authors:** 


### RESIDENT MEDICAL OFFICERS' DEPARTMENT.

### THE R.M.O. AND OUTSIDE EMERGENCY CALLS.

We have received the following communication from a reader :—

This matter raised by your correspondent in The Hospital of December 12 is of great importance. As a former R.M.O. perhaps the writer may be permitted to make a few remarks on the subject.

In the rules of some hospitals it is specifically laid down that no work except that in connection with the hospital is to be undertaken by the R.M.O. If that rule is to be strictly kept then the R.M.O. is definitely debarred from responding to outside calls. By subscribing to that rule a R.M.O. could hardly be blamed. Yet there are some people who would at once bring forward an accusation of inhumanity against him if he refused to go.

When no definite rule regarding the question is included in the hospital by-laws it is more difficult to decide what to do. Granting that there is an unwritten law, is this binding in such cases? It is symbolical of the medical profession that its members are always ready and willing to give prompt assistance in case of need, and it would only be following this custom if the R.M.O. offered his services. It surely would be unreasonable for a hospital committee to censure the R.M.O. for such an action, provided that the hospital work had not been neglected.

The nature of the case might influence the R.M.O.'s action. For instance, if the case were one of hanging or of a foreign body impacted in the windpipe prompt action is necessary, and the R.M.O. might decide to go. If on the other hand the case were one of, say, opium poisoning or of convulsions in a child, the R.M.O. might offer to see and treat the case if brought to the hospital. It must, however, be admitted that the information brought is often very vague, and it is also true that the message brought is sometimes very much exaggerated. The R.M.O. must use his discretion, because many cases which appear urgent to the minds of the patient's friends are really not so. In the absence of any rule forbidding it, probably the R.M.O. should respond to an outside emergency call if not engaged with any serious case in hospital.

If the R.M.O. has responded to a call, what should he do when another medical man arrives? The ethical course would seem to be for him to hand over the case to the practitioner, for in reality he has been acting as a substitute. As many of these cases are of the poorer classes the practitioner may order removal to hospital (provided, of course, any further treatment is necessary), and so the R.M.O. may in the end have the case under his charge.

Hospital committees generally introduce rules forbidding outside work for the purpose of preventing their residents engaging in private practice. They appear to frame such by-laws in order to protect their interests and so that the work of their institution may not be neglected. It is thus possible that many committees would not construe response to an emergency call (which indicates that the case is a critical one) as breach of contract.

